# Phenotypic characterisation of *Saccharomyces* spp. yeast for tolerance to stresses encountered during fermentation of lignocellulosic residues to produce bioethanol

**DOI:** 10.1186/1475-2859-13-47

**Published:** 2014-03-27

**Authors:** Tithira T Wimalasena, Darren Greetham, Marcus E Marvin, Gianni Liti, Yogeshwar Chandelia, Andrew Hart, Edward J Louis, Trevor G Phister, Gregory A Tucker, Katherine A Smart

**Affiliations:** 1 Bioenergy & Brewing Science, School of Biosciences, Sutton Bonington Campus, University of Nottingham, Loughborough, Leicestershire LE12 6RD, UK; 2 Centre for Genetic Architecture of Complex Traits, Department of Genetics, University of Leicester, Adrian Building, Leicester LE1 7RH, UK; 3 Institute for Research on Cancer and Aging, Faculty of Medicine, 28, Avenue De Valombrose, 06107 Nice, Cedex-02, France; 4 Current address: SABMiller plc, SABMiller House. Church Street West, Woking, Surrey GU21 6HS, UK; 5 Pepsico Int, 4, Leycroft Road, Leicester LE4 1ET, UK

**Keywords:** *Saccharomyces* spp., Phenotypic microarray, Bioethanol, Fermentation

## Abstract

**Background:**

During industrial fermentation of lignocellulose residues to produce bioethanol, microorganisms are exposed to a number of factors that influence productivity. These include inhibitory compounds produced by the pre-treatment processes required to release constituent carbohydrates from biomass feed-stocks and during fermentation, exposure of the organisms to stressful conditions. In addition, for lignocellulosic bioethanol production, conversion of both pentose and hexose sugars is a pre-requisite for fermentative organisms for efficient and complete conversion. All these factors are important to maximise industrial efficiency, productivity and profit margins in order to make second-generation bioethanol an economically viable alternative to fossil fuels for future transport needs.

**Results:**

The aim of the current study was to assess *Saccharomyces* yeasts for their capacity to tolerate osmotic, temperature and ethanol stresses and inhibitors that might typically be released during steam explosion of wheat straw. Phenotypic microarray analysis was used to measure tolerance as a function of growth and metabolic activity. *Saccharomyces* strains analysed in this study displayed natural variation to each stress condition common in bioethanol fermentations. In addition, many strains displayed tolerance to more than one stress, such as inhibitor tolerance combined with fermentation stresses.

**Conclusions:**

Our results suggest that this study could identify a potential candidate strain or strains for efficient second generation bioethanol production. Knowledge of the *Saccharomyces* spp. strains grown in these conditions will aid the development of breeding programmes in order to generate more efficient strains for industrial fermentations.

## Background

It is generally recognised that ‘renewable’ forms of energy, such as those generated from lignocellulosic biomass, will become increasingly important. Currently the production of liquid biofuels by fermentation has focussed on the conversion of hexose sugars to form bioethanol [1], where hexose carbohydrates are released from sucrose in crops such as sugar cane [2,3], or from starch. However, use of these biomass resources has met criticism and an increased interest in non-starch or cane biomass conversion [4,5].

Bioethanol produced from lignocellulosic residues may be more socioeconomically prudent, however, the technical block to this approach remains the efficient conversion of hexose and pentose sugars into ethanol or alternative liquid biofuels [6]. *Saccharomyces cerevisiae* is widely employed for the commercial production of bioethanol from hexose sugars. However, *S. cerevisiae* strains are unable to efficiently utilise pentose sugars [7], despite containing a xylose utilisation pathway [8] and an arabinose metabolic pathway [9]. This restricts the potential use of non-recombinant strains of *S. cerevisiae* for the production of bioethanol from lignocellulosic feedstocks [10-13].

*Saccharomyces* spp. are attractive because of their capacity to produce ethanol. Their relatively high tolerance to osmotic stress and ethanol and their tolerance to anaerobic conditions are characteristics that are suitable for large-scale fermentation [14]. Much less is known about their capacity to tolerate the inhibitors released during the formation of lignocellulosic hydrolysates [15,16]. The *Saccharomyces* spp. (formerly termed *Saccharomyces sensu stricto*) complex consists of seven closely related but distinct species; *S. cerevisiae, S. paradoxus, S. mikatae*, *S. kudriavzevi S. arboricolus,* and *S. uvarum,*[17-20]. Within *S. cerevisiae* there is an enormous amount of genetic variability that is believed to result from its geographical movement by man along with outcrossing to generate strains with mosaic genomes [21,22]. Five clean lineage strains (West African, Wine European, Sake, North American and Malaysian) of *S. cerevisiae* that are representative of specific genomic clades have been identified [22] and engineered to enable genetic tractability [23-25].

Research has shown that approaches such as phenotypic selection of natural isolates, breeding programmes assisted by technological tests, quantitative trait locus (QTL) introgression and genetic engineering have been successful in strain development for the food and beverage industries [1,26,27]. In addition, interbreeding is very common among *Saccharomyces* strains giving rise to naturally occurring novel hybrid strains that have been identified in the brewing and wine industries [28,29]. All species within the *Saccharomyces* spp. complex can be mated to form hybrid diploids and these can subsequently be utilised to produce bioethanol. Therefore, phenotypic screening of *Saccharomyces* strains can be an important initial tool for isolating a strain with desirable traits for efficient bioethanol fermentation.

Here, we have screened *Saccharomyces* spp. strains and selected hybrids for phenotypic variation in terms of tolerance to osmotic stress, temperature (30°C-40°C), increased ethanol concentration and inhibitory compounds released through the pre-treatment of lignocellulosic biomass and utilisation of hexose and pentose sugars. By coupling phenotypic and genetic analysis, selective breeding and evolutionary engineering, novel yeast strains can be produced with inherent properties for improving industrial processes such as bioethanol production from lignocellulosic wastes [30-32].

The aim of this study was to identify strains capable of tolerating the stress and inhibitor conditions associated with lignocellulosic bioethanol fermentation. A selection of *Saccharomyces* strains were chosen that had been isolated from natural habitats, wine, beer, baking or clinical backgrounds [22].

## Results

### Phenotypic variation and ranking of responses of yeast strains to stress

Ninety strains of *Saccharomyces* spp. (89 formerly termed *Saccharomyces sensu stricto* yeast and the phylogenetically distinct *S. castelli sensu stricto* group outsider) were screened. Phenotypic responses to parameters encountered during the fermentation of lignocellulosic hydrolysates were tested. These included the utilisation of hexose and pentose sugars, resistance to conditions within bioreactors, such as osmotic, ethanol and temperature stress and resistance to phenolic and aromatic inhibitory compounds formed during the steam explosion of lignocellulosic waste. Phenotypic variation of the strains to these stresses was observed and ranked according to the impact on metabolic output, defined here as percentage of redox signal intensity of control (Additional file 1: Figure S1).

### Utilisation of hexose and pentose sugars by *Saccharomyces* spp. strains

**Figure 1:**
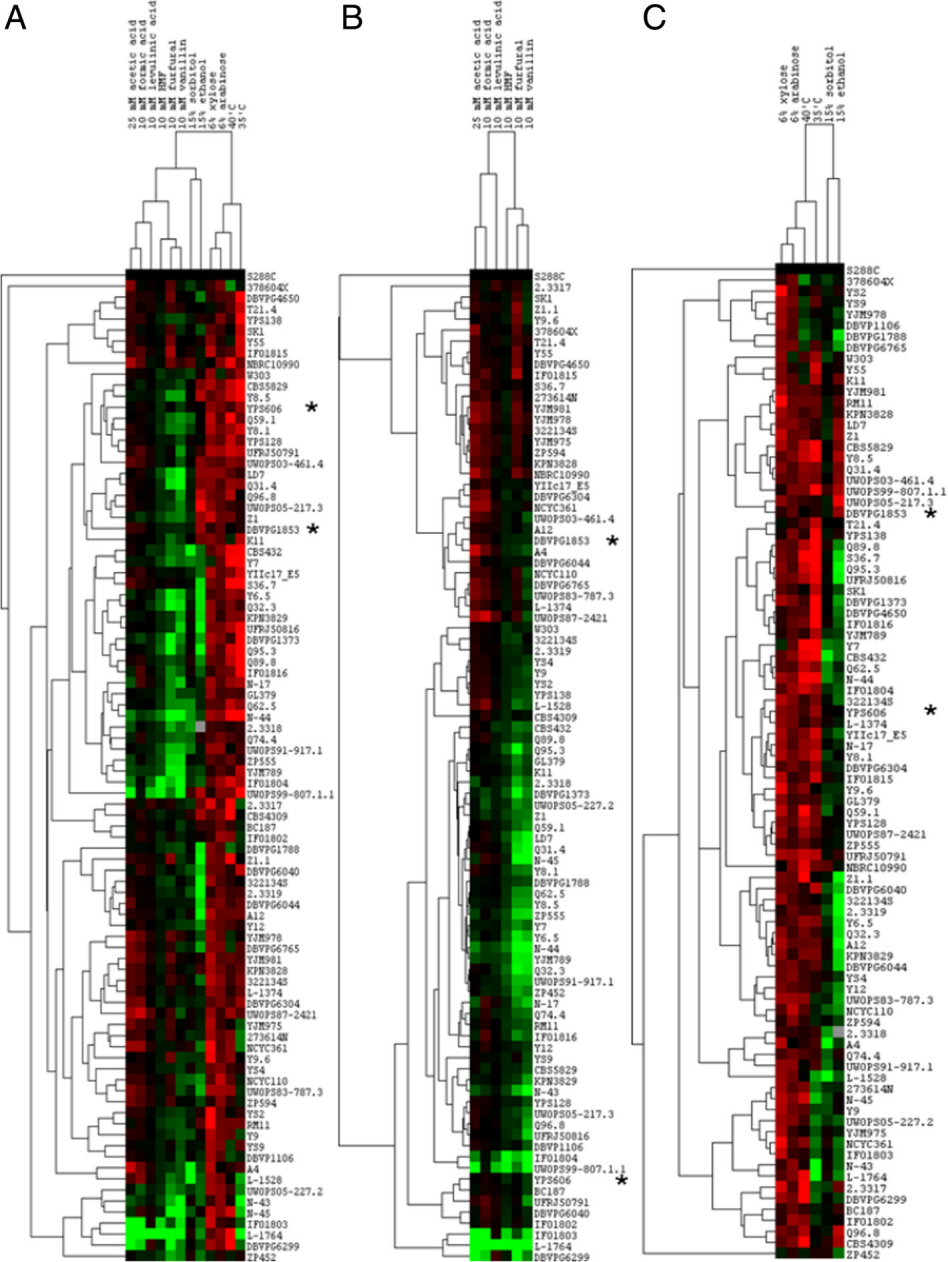
**Cluster analysis of combined yeast data sets.** Data was drawn from metabolic output under stress conditions for 90 *Saccharomyces* spp. All measurements were compared against a reference strain S288C. The full colour range represents log ratios of −3.0 to 3.0. Phenotypes were quantified using high-resolution micro- cultivation measurements of population density. Strain (n = 2) doubling time (rate) phenotypes in relation to the S288C are displayed. Green = poor metabolic output, red = good metabolic output. Hierarchical clustering of phenotypes was performed using a centred Pearson correlation metric and average linkage mapping. **(A)** Cluster data based on maximal sugar utilisation rates under stress conditions, **(B)** cluster data based on maximal sugar utilisation rates under inhibitor stress, **(C)** cluster data based on maximal sugar utilisation rates under fermentation stress conditions.

Hydrolysates derived from LCM contain hexose and pentose sugars, [33], metabolic output (defined here as the detection of conversion of a redox sensitive dye from an oxidised to reduced state) on glucose, xylose and arabinose was measured. These sugars are the most abundant in LCM hydrolysates after pre-treatment of lignocellulose [34]. There was measurable metabolic output when utilising glucose for all strains in this study with variation observed between the strains (35–75 redox signal intensity) after 48 hours incubation (Additional file 2). Assays for utilisation of the pentose sugars, xylose and arabinose, revealed that the majority of the strains exhibited very poor metabolic output (<20 redox signal intensity) after 50 hrs incubation (Additional file 1: Figure S1). The reference yeast used for these experiments, S288C, has previously been reported as being a poor pentose utilising yeast [12] and we failed to observe significant improvements in metabolic output when utilising either xylose or arabinose (Additional file 1: Figure S1). There are a few strains which to a degree exhibited metabolic output when utilising xylose such as *S. cerevisiae* YS2, or when utilising arabinose such as *S. cerevisiae* NCYC110 (Figure 1A and 1C). However, assays comparing these strains with known pentose utilisation yeast (*Candida shehatae* or *Scheffersomyces spitis*) highlighted the poor pentose utilisation by *Saccharomyces* spp. (data not shown). *Saccharomyces* spp. vary in their biochemical profiles [35] and variation in metabolic output in general may have accounted for some of the differences in xylose utilisation between strains. However, there was no correlation observed between glucose utilisation and xylose utilisation (R = 0.081). *Saccharomyces* spp. strains shared similarities in the utilisation of xylose and arabinose (R = 0.41). However, the majority of the strains analysed showed a lack of significant xylose or arabinose utilisation.

### Phenotypic variation to stress inherent to fermentations within *Saccharomyces spp.* strains

**Figure 2:**
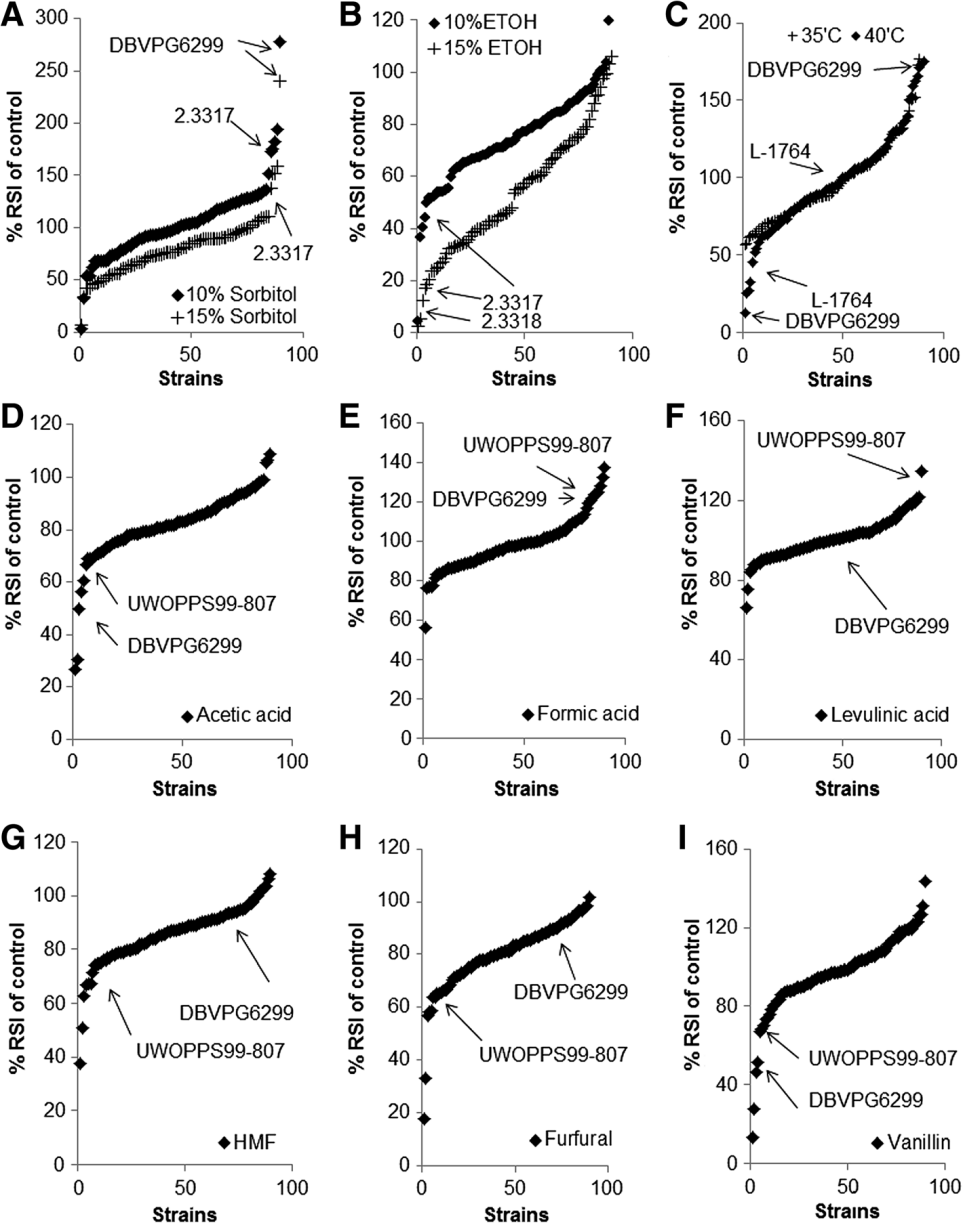
**Phenotypic microarray analysis (redox signal intensity) of *Saccharomyces *spp. for the effect of fermentation stress (temperature, osmotic and ethanol) and inhibitors at 50 hrs.** Yeast were grown in minimal medium containing 6% glucose at 30°C and metabolic activity assessed at 50 hrs time point. Results are plotted as% of RSI (redox signal intensity) where metabolic output in the presence of defined stresses were compared to unstressed conditions. **(A)** 10% and 15% sorbitol, **(B)** 10% and 15% ethanol, **(C)** temperature (35°C and 40°C) **(D)** 25 mM acetic acid, **(E)** 10 mM formic acid, **(F)** 10 mM levulinic acid, **(G)** 10 mM furfural, **(H)** 10 mM HMF and **(I)** 10 mM vanillin, on sugar utilisation on *Saccharomyces* spp. ranked from sensitive to tolerant strains. Selected yeast strains have been highlighted as appropriate.

Constant exposure to high temperatures or severe osmotic stress has been used to identify tolerant yeast strains [36]. Tolerance to osmotic, ethanol or temperature stress was characterised by profound phenotypic variation between yeast strains (Figure 1A-C). Using the ranking system, *S. uvarum* (DBVPG6299), *S. paradoxus* (Q74.4) and *S. arboricolus* (2.3317) strains were identified as being osmotically tolerant when compared with other strains used in the study (Figure 2A and Table 1). In general, *S. kudriavzevii* spp. displayed higher sensitivity to osmotic stress when compared with other *Saccharomyces* spp. yeast strains.

**Table 1:** Ranking of yeast strains for tolerance to stress conditions in bioethanol fermentation

**Sorbitol**			**Ethanol**		**35′C**		**40′C**		**Acetic acid**		**Formic acid**		**Furfural**		**HMF**		**Vanillin**		**All stress**	
**Sensitive**	*S. p*	N-17	*S. a*	2.3318	*S. c*	DBVPG1853	*S. p*	CBS432	*S. a*	2.3317	*S. c*	Y12	*S. c*	Y12	*S. cas*	CBS4309	*S. c*	Y12	*S. c*	DBVPG1853
	*S. c*	W303	*S. a*	2.3317	*S. c*	UWOPS83-787.3	*S. k*	NBRC10990	*S. c*	CBS4309	*S. p*	N-17	*S. cas*	CBS4309	*S. c*	Y12	*S. cas*	CBS4309	*S.u*	L-1764
	*S. k*	ZP452	*S. p*	LD7	*S. c*	UWOPS03-461.4	*S. b*	DBVPG6045	*S. c*	Y12	*S. c*	YJM981	*S. p*	N-45	*S. c*	322134S	*S. a*	2.3317	*S. p*	Q32.3
	*S. c*	DBVPG1788	*S. p*	UFRJ50816	*S. c*	Y9	*S. a*	2.3317	*S. p*	Q74.4	*S. c*	YIIc17_E5	*S. p*	Q74.4	*S. m*	IFO1815	*S. p*	Q74.4	*S. p*	UFRJ50816
	*S. p*	Q32.3	*S. p*	Q32.3	*S. p*	Y7	*S. b*	L-1764	*S. b*	UWOPS99-807.1.1	*S. cas*	CBS4309	*S. c*	YIIc17_E5	*S. c*	YIIc17_E5	*S. c*	YIIc17_E5	*S. c*	UWOPS83-787.3
**Tolerant**	*S. c*	YJM789	*S. c*	YPS606	*S. c*	DBVPG6040	*S. c*	YS2	*S. b*	DBVPG6045	*S. c*	UWOPS83-787.3	*S. c*	RM11	*S. c*	DBVPG6040	*S. c*	Y9	*S. p*	UWOPS91-917.1
	*S. c*	SK1	*S. p*	Q96.8	*S. p*	Q96.8	*S. p*	Q89.8	*S. p*	YPS138	*S. b*	L-1764	*S. a*	2.3317	*S. p*	Y6.5	*S. p*	GL379	*S. p*	Q89.8
	*S. b*	UWOPS99-807.1.1	*S. c*	YJM975	*S. c*	378604X	*S. p*	UWOPS91-917.1	*S. m*	IFO1816	*S. c*	S288C	*S. c*	YJM978	*S. c*	YJM978	*S. c*	YPS606	*S. c*	YPS606
	*S. c*	378604X	*S. b*	ZP555	*S. p*	IFO1804	*S. c*	YJM975	*S. p*	UFRJ50816	*S. c*	YJM978	*S. b*	L-1764	*S. c*	YS9	*S. c*	S288C	*S. c*	378604X
	*S. p*	DBVPG6304	*S. c*	Y12	*S. a*	2.3317	*S. c*	DBVPG6044	*S. p*	Y8.1	*S. k*	IFO1803	*S. c*	YPS128	*S. c*	L-1374	*S. c*	UWOPS87-2421	*S. c*	YJM975
	*S. c*	*S. cerevisiae*																		
	*S. p*	*S. paradoxus*																		
	*S. k*	*S. kudriavzevii*																		
	*S. u*	*S. uvarum*																		
	*S. a*	*S. arboricolus*																		
	*S. cas*	*S. castelli*																		
	*S. m*	*S. mikatae*																		

Ranking of *Saccharomyces* spp. strains for stress conditions analysed in this study. The five most tolerant and sensitive strains for each stress conditions are shown in this table. The full list of strains is available in Additional file 1: Figure S1.

Screening for tolerance to 15% ethanol, six of the ten most tolerant strains (determined by observable metabolic output under stress conditions when compared with other yeast strains in this study) were *S. cerevisiae* strains isolated from either a clinical or a fermentation background. *S. arboricolus* strains (2.3317 and 2.3318) displayed the highest sensitivity to ethanol when compared with other yeast in this study (Figure 2B), however, 2.3317 is tolerant to osmotic (sorbitol) stress when compared with the other *S. arboricolus* strains (Figure 1A).

For temperature stress there was a reduction in metabolic output at 40°C (using data from assays at 30°C as a control) for the majority of strains when compared with output at 30°C and 35°C. Interestingly, most of the *S. cerevisiae* isolated from either a clinical or baking backgrounds displayed tolerance to 40°C. However, *S. kudriazevii* strains (with the exception of strain IFO1803) were sensitive to increasing temperature; they displayed a reduction in metabolic output at 35 and 40°C when compared with 30°C.

The metabolic output at 35°C was increased for *S. uvarum* strains (DBVPG6299 and UWOPS99-807.1.1) used in this study. The metabolic output of the *S. uvarum* strain (L1764) was decreased at 35°C compared to 30°C. Growth analysis of these strains at 35°C and 40°C confirmed the results obtained from PM analysis (Additional file 1: Figure S1).

### Phenotypic variation to inhibitors within *Saccharomyces* spp. strains

The presence of inhibitors in lignocellulosic fermentations has been shown to have a profound effect on the sugar utilisation and viability of *S. cerevisiae* (Greetham, *et al*., unpublished data). *Saccharomyces* spp. were assayed for the effect of acetic acid, formic acid, levulinic acid, HMF, furfural and vanillin on metabolic output using inhibitory concentrations. The concentrations tested for acetic acid, furfural and formic acid have been shown to be released during pre-treatment of lignocellosic material [34] and for HMF, vanillin and levulinic acid have been previously shown to inhibit yeast [37,38] (Greetham *et al*., unpublished).

In general, *S. uvarum* strains, such as (DBVPG6299), screened in this study were sensitive to inhibitory compounds. However, there were exceptions such as UWOPS99-807.1.1, which exhibited tolerance to both formic acid and levulinic acid (Figure 2E and 2F). Furfural tolerant strains were either *S. cerevisiae* or the closely related *S. paradoxus* (Figure 2D-2I). Therefore, the general trends in tolerance/sensitivity followed species designations.

### Hierarchical clustering followed species and trait boundaries

Utilising a phenotypic microarray assay, differences between yeast species in terms of tolerance to stresses inherent to fermentations have been defined. Defining tolerance to more than one stress has allowed for the identification of robust yeast for future bioethanol fermentations (Table 1). However, the data was analysed further in an attempt to discern whether phenotypic response to stress is general, or whether or not specific yeast clades cluster together. Hierarchical clustering of *Saccharomyces* spp. based on phenotypic trait profiles (Figure 1A and 1B) was compared with the haploid reference laboratory strain *S. cerevisiae* S288C. The reference strain displayed sensitivity to stresses inherent to fermentations and hence the majority of strains in the cluster analysis appeared red (more tolerant) (Figure 1A and 1B). The reference yeast, S288C is one of the more sensitive strains to the presence of inhibitory compounds when compared with other yeast analysed in this study. However, it was one of the more thermo-tolerant strains (displaying metabolic activity at 35 or 40°C when compared with metabolic output at 30°C) and was phenotypically distinct from the other yeast in response to stress (Figure 1A-C). S288C is known to be phenotypically distinct from other *S. cerevisiae* strains and this has been previously reported [39].

The phenotypic microarray results for pentose sugars clustered with the results for temperature, ethanol or osmotic stress (Figure 1A and 1C). On the other hand, response to inhibitory compounds clustered separately from the phenotypic response to fermentation stress (osmotic, temperature and ethanol). Inhibitory compounds (such as weak acids or furanic compounds) clustered together (Figure 1A and 1B).

There was a clearly observed dichotomy at the UWOPS99-807.1.1/2.3317 boundary (Figure 1A). Strains S288C to UWOPS99-807 are characterised by similar phenotypic responses to inhibitory compounds (with the exception of formic acid), are sensitive to ethanol and displayed better thermo-tolerance than the reference strain. Strains 2.3317 to DBVPG1106 were characterised as being more tolerant to inhibitory compounds, osmotic stress and temperature stress than S288C, but more sensitive to the presence of ethanol. Strains on Figure 1A from A4-ZP452 are inhibitor (including formic acid) and/or temperature sensitive.

We also observed a boundary between weak acid tolerance/furanic/phenolic tolerance and weak acid/furanic/phenolic sensitivity (Figure 1B). Strains from 2.3317 to W303 were more tolerant to the presence of weak acids than those from W303 to DBVPG6299. However, strains W303 to DBVPG6299 were more sensitive to the presence of furanic or phenolic compounds than the reference strain, S288C.

However, *S. uvarum* and *S. kudriavzevii* clustered together when the effect of stress on was determined, they share similar responses to inhibitor and temperature stress (Figure 1A and 1B).

### Confirmation of phenotypic microarray strain assessments using mini-fermentation analysis

The objective of this study was to identify potential candidate strains for efficient second generation bioethanol fermentations. Phenotypic variation to stress as mentioned earlier was ranked according to tolerance to the various stresses, allowing the identification of tolerant yeast for any tested stress condition (Table 1). From the 90 strains screened and ranked *S. cerevisiae* strain, YPS 606, was selected as a strain with general tolerance to stress with the *S. cerevisiae* strain, DBVPG1853, is the most sensitive strain after 48 hours of the assay (Additional file 1: Figure S2). The latter is also phenotypically similar to S288C, which is also sensitive to stress (Figure 1A-C).

**Figure 3:**
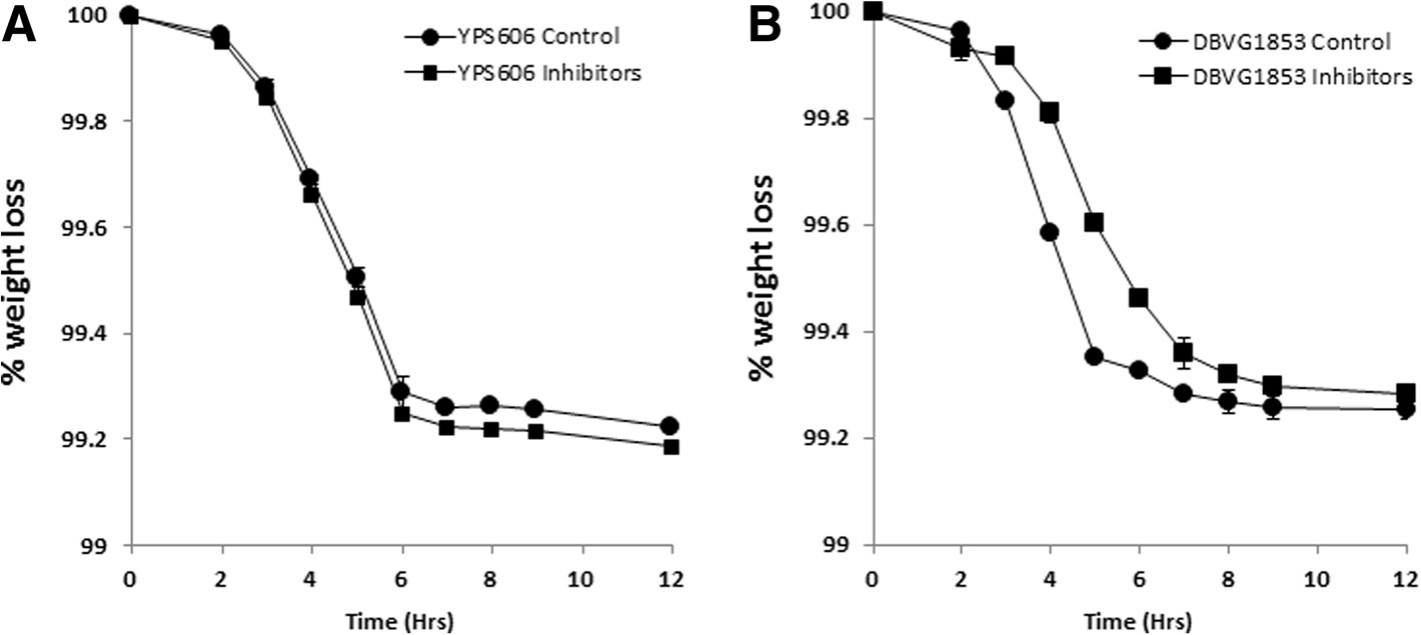
**Performance of yeast strains during fermentations in the presence of inhibitory compounds.** Comparison of sensitive (DBVPG1853) and tolerant (YPS606) *S. cerevisiae* strains to stress, fermentation kinetics analysis using mini-fermenters on 4% (w/v) glucose and inhibitors (10 mM acetic acid, 5 mM formic acid, 5 mM levulinic acid, 5 mM HMF, 5 mM furfural and 5 mM vanillin in combination) at 35°C using **(A)***S. cerevisiae* YPS606 and **(B)** DBVPG1853. Each data point represents mean value of biological triplicate experiments with SD error bars.

These two strains were used in fermentation experiments, with fermentation progression monitored by measuring weight loss over time. This has been shown to correlate with sugar utilisation [40] in the presence of inhibitory compounds. These were compared to fermentations of unstressed controls at 35°C. Fermentations with YPS606 were not affected by the presence of inhibitors when compared with controls (Figure 3A). However, presence of inhibitory compounds did slow a fermentation using DBVPG1853 by approximately 2 hrs when compared with unstressed controls (Figure 3B).

### Trait hereditary in diploid hybrids confers tolerance compared with parental sensitivity

*Saccharomyces* strains (YPS128, Y12, Y55 and IFO1803) were used to generate diploid hybrids. By assessing tolerance to stress, it was observed that YPS128 and Y12 exhibited different sensitivities to the presence of inhibitory compounds (defined as the number of more tolerant yeast strains assayed for in this study). Y55 (*S. cerevisiae*) and IFO1803 (*S. kudriavzevii*) exhibit different tolerance to the presence of inhibitory compounds, temperature stress, or ethanol stress (Additional file 1: Figure S3 and Additional file 2). These hybrid strains and their homozygous parents were then assessed for tolerance to a range of fermentation stress conditions using PM assays.

**Figure 4:**
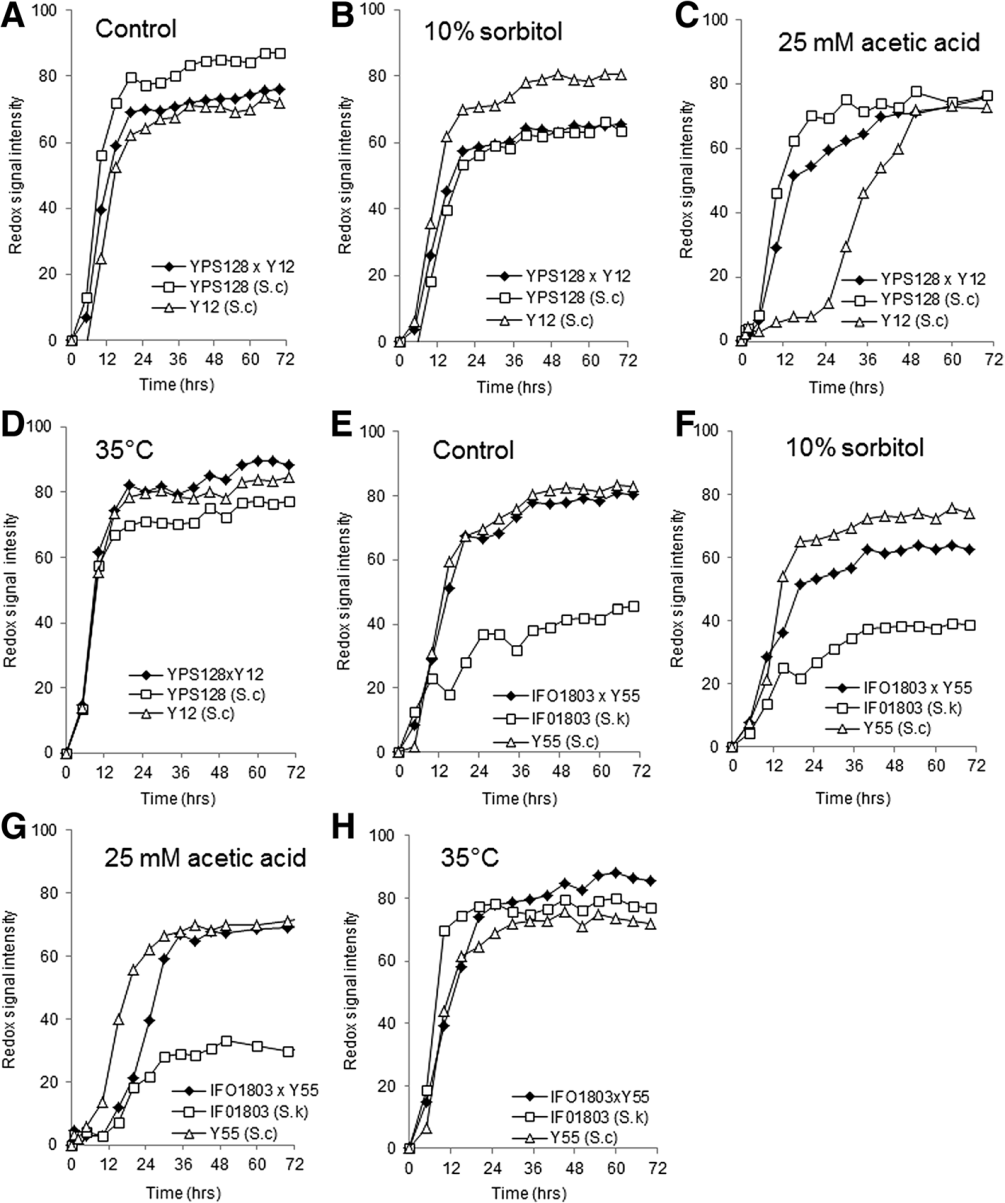
**Phenotypic microarray analysis (redox signal intensity) of hybrids.** The response of hybrid diploids to stress was compared with the parental stains *S. cerevisiae* (Y21) and *S. cerevisiae* (YPS128) **(A-E)** and *S. cerevisiae* (Y55) and *S. kudriavzevii* (IFO1803) (F-I) Metabolic output for *S. cerevisiae* (Y12), *S. cerevisiae* (YPS128) and a diploid cross (Y12 × Y128) for **(A)** control (6% (w/v) glucose at 30°C), **(B)** 6% glucose and 10% (w/v) sorbitol, **(C)** 6% glucose and 25 mM acetic acid **(D)** 6% glucose at 35°C. Metabolic output for *S. cerevisiae* (Y55) and *S. kudriavzevii* (IFO1803) and a diploid cross (Y55 × IFO1803) for **(E)** control (6% (w/v) glucose at 30°C), **(F)** 6% glucose and 10% (w/v) sorbitol, **(G)** 6% glucose and 25 mM acetic acid and (H) 6% glucose at 35°C. The data shown is an average of triplicate values with standard deviations shown.

The tolerances of a *S. cerevisiae* cross between YPS 128 and Y12 to formic acid, osmotic stress and high temperature was measured. Under unstressed control conditions, metabolic output of the heterozygous hybrid diploid (YPS 128 × Y12) was an intermediate of the metabolic output of either haploid parent (Figure 4A). There was a variation in the phenotypic response to stress observed when compared with either parent. When under osmotic or temperature stress, the hybrid diploid had the same profile as the sensitive parent Y12 (Figure 4B and 4D). Y12 was highlighted as being sensitive to the presence of inhibitory compounds in the initial screen, whereas YPS128 had a more tolerant phenotype (Additional file 1: Figure S1). The hybrid diploid was more tolerant to stress induced by inhibitory compounds than Y12 (Figure 4C).

We wanted to observe how a heterozygous hybrid diploid resulting from a cross between a non-*S. cerevisiae* strain that was sensitive to stress (*S. kudriavzevii* 1803) and a stress tolerant *S. cerevisiae* strain (Y55) would behave under our test conditions. It was observed that under unstressed control conditions, the hybrid diploid displayed an identical metabolic output to the parental strain Y55 (Figure 4E). The hybrid diploid was more sensitive to osmotic or acetic acid induced stress than Y55, but was more tolerant than the *S. kudriavzevii* strain 1803 (Figure 4F and 4G). There was little variation observed under temperature stress between the parents and hybrid (Figure 4H).

## Discussion

Industrial scale fermentations always favour yeast with efficient fermentation capabilities, particularly *S. cerevisiae* and the closely related *S. uvarum*[41]. Yeast cells encounter osmotic stress due to high sugar and solute concentrations at the beginning of fermentation [42]. During fermentation additional stresses such as the accumulation of ethanol also become relevant [43]. In addition, during second generation bioethanol fermentations organisms are exposed to inhibitory compounds released by the pre-treatment of lignocellulosic material. Complex and advanced strategic approaches, based on genetic engineering strategies have been utilised to increase the innate tolerance of yeast cells to inhibitory compounds [7]. Understanding the mechanisms of resistance to these compounds are commercially attractive [44]. The possibility of using natural selection and breeding programmes to develop non-GM strains of *S. cerevisiae* that can efficiently convert hexose and pentose sugars into ethanol under stressful fermentation conditions is being pursued [45] and is ecologically and ideologically attractive.

Screening for xylose utilisation within the *Saccharomyces* spp. identified strains that have been previously reported as being better utilisers of xylose [12]. In addition *S. kudriavzevii*, strains have been previously reported as being temperature sensitive [26,46]. However, *S. cerevisiae* and *S. paradoxus* strains were also identified that were able to tolerate high osmotic stress (15% sorbitol), high ethanol concentrations (15% ethanol) and higher temperatures (35°C and 40°C). Assays at 35°C were characterised by an increase in metabolic output for the majority of *S. cerevisiae* and *S. paradoxus* strains. An increase in metabolic output at 35°C for *S. cerevisiae* has been reported previously [26,46]. It was observed that *Saccharomyces* strains isolated from clinical backgrounds exhibited the highest tolerance to thermal stress as may be expected. *S. uvarum* has been identified as thermo-sensitive [47]. However, metabolic output was increased for *S. uvarum* strains (DBVPG6299 and UWOPS99-807.1.1) at 35°C. Metabolic output for DBVPG6299 was significantly reduced at 40°C and this yeast has been previously reported as being temperature sensitive [48]. The metabolic output of the *S. uvarum* strain (L1764) decreased at 35°C when compared with metabolic output at 30°C. The phylogenetically distinct *S. castelli*[49] was observed to be thermo-tolerant characterised by increased metabolic output at 40°C when compared with other yeast.

Tolerances/sensitivities tended to follow strain designations, for example all *S. cerevisiae* and *S. paradoxus* strains investigated in our study were able to tolerate 10% ethanol. However, *S. cerevisiae* isolated from beverage fermentations were the most tolerant to 15% ethanol. *S. cerevisiae* strains from fermentation backgrounds encounter higher alcohol concentrations, particularly sake fermentations which can generate at least 20% (v/v) ethanol [50]. In contrast, *S. arboricolus* and *S. kudriavzevii* strains were identified as sensitive to ethanol, supporting reports that these strains were isolated from natural environments and never exposed to an industrial fermentative environment [20,26]. In general, yeast isolated from clinical or fermentation backgrounds displayed the highest tolerance to stresses inherent to fermentations. *S. arboricolus*[20] displayed tolerance to the presence of weak acids when compared with other yeast assayed in this study, however, this yeast sensitive to the toxic effects of ethanol, very little has been published on this yeast and findings here suggest it has some properties which may be relevant for improving bioethanol fermentations.

Clustering revealed that phenotypic response to weak acids, furans, pentose use, osmotic, ethanol and temperature stress all clustered separately and there was an overall strain variation within the *Saccharomyces* spp. to stress (Figure 1A-1C).

*S. cerevisiae* strains have been identified as the yeast of choice for efficient bioethanol fermentation due to their ability to convert hexose sugars to high concentrations of ethanol despite the presence of inhibitory compounds in the medium [51]. Using the phenotypic microarray screen, yeast with differing tolerances to stresses were selected and assessed for performance during fermentation. Fermentation profiles correlated with profiles from the phenotypic microarray screen. Thus, tolerant yeast (YPS606) was not inhibited by the presence of an inhibitor stress when compared with control conditions. Interestingly, fermentations using more sensitive yeast (DBVPG1853) under inhibitor stress were characterised by a two hour “slow down” when compared with control.

Hybrid yeast strains are the next step in development of industrial bioethanol yeast, the phenotypes of diploid hybrids generated using selected *Saccharomyces* spp. strains (S.c.YPS128 × S.cY12; S.c.Y55 × S.k.IFO1803) was compared to the metabolic performance of parents under stress conditions. Hybrid strains displayed tolerances similar to one or the other parents or in some instances outperformed either parent. Hybrids displaying tolerance above that of either parent has been noted previously in hybrid diploids [52]. Assessing a cross between *Saccharomyces* spp. (*S. kudriavzevii* 1803 x *S. cerevisiae* Y55), we observed that this diploid was as metabolically active as one of the parents. This hybrid diploid was phenotypically similar to the more tolerant parent under stress conditions, highlighting the potential for cross-breeding between different *Saccharomyces* spp. for desirable bioethanol strains. Our approach has shown that selected hybrid formation can be a useful tool for the development of novel strains tolerant to the stresses present in bioethanol fermentations.

## Conclusions

In this study, the responses of *Saccharomyces* spp. strains to the stresses present during bio-ethanol fermentations have been characterised. Considerable natural variation was observed in the stress responses of these yeast and many strains are tolerant to multiple inhibitors. These findings were confirmed when fermentation profiles of tolerant and sensitive yeast were compared. Some of the strains (YPS606 S.c.; DVBPG6040-S.c.) identified in the study displayed useful phenotypes, such as the tolerance to most of the stress conditions identified in second generation bioethanol fermentations. Hybrids can exhibit more desirable properties than their original parents, an important parameter for future strain development *via* breeding programmes. Knowledge of the *Saccharomyces* strains grown in these conditions will aid the development of breeding programmes in order to generate more efficient strains for industrial fermentations. Use of this data will aid increased economic output and the establishment of a viable second-generation bioethanol industry.

## Methods

### Yeast strains and growth conditions

Many of the *Saccharomyces* spp. strains used in this study have been previously described [22,53,54]. Additional isolates tested included two strains of *S. arboricolus*[20,55,56]. The majority of the strains analysed in this study were wild isolates [22] that do not contain any gene deletions or auxotrophic markers. The homothallic parent strain of each isolate had been sporulated and the resultant spores dissected and allowed to self-mate to generate homozygous diploids before they were sequenced [22]. Selected representatives of clean lineage *S. cerevisiae* strains were made genetically tractable by deleting HO and creating an *URA3* auxotrophy by deletion [23] Haploids of these were crossed to form heterozygotes with auxotrophic markers as described previously [23,24]. The hybrid *S. kudriavzevi* 1803 × *S. cerevisiae* Y55 was generated previously [57] and was also utilised for this study.

For vegetative growth, either yeast extract peptone dextrose (YPD) medium [1% yeast extract (Oxoid); 2% (w/v) Bacto-peptone (Oxoid); 2% (w/v); 2% (w/v) glucose], or YNB (Yeast nitrogen base) medium [0.67% (w/v) YNB with amino acids and ammonium sulphate; 6% (w/v) glucose] were used. For analysis of growth using alternative carbon sources (pentose sugars), glucose was replaced with 2% xylose. Cultures were cryopreserved in 20% glycerol at −80°C. Most strains can be obtained from the National Collection of Yeast Cultures (NCYC; see http://www.ncyc.co.uk/ for information). All isolates were stored at −80°C in a 96-well plate format in 20% glycerol. More detailed information on each strain and species can be found in the Additional file 1: Figure S1.

### Phenotypic microarray analysis

Phenotypic microarray (PM) technology (Biolog, US) assay is based on the detection of metabolic output using a reporter system [58], the reporter system utilises a redox sensitive tetrazolium dye which upon reduction correlates with an increase in metabolic rate of a cell which is oxidizing a carbon source. For PM analysis each individual well contained growth medium consisting of a final concentrations of 0.67% (w/v) yeast nitrogen base (YNB) and 6% (w/v) glucose as appropriate for the final assay volume of 120 μl, supplemented with 2.6 μl of yeast nutrient supplement mixture (NS × 48- 24 mM adenine-HCl, 4.8 mM L-histidine HCl monohydrate, 48 mM L-leucine, 24 mM L-lysine-HCl, 12 mM L-methionine, 12 mM L-tryptophan and 14.4 mM uracil) and 0.2 μl of dye D (Biolog, Hayward, CA, USA). The final volume was made up to 30 μl using sterile distilled water. This was made up fresh as a stock sufficient for each experiment and 30 μl dispensed to individual microtitre plate wells containing increasing concentrations of the appropriate inhibitors (acetic acid, formic acid, furfural, hydroxy-methyl furfural (HMF), levulinic acid and vanillin). Stock solutions (1 M) of aliphatic weak acids such as acetic acid, formic and levulinic acid were prepared using reverse osmosis (RO) sterilised water; furfural, HMF and vanillin were prepared as 1 M stock solutions in 100% ethanol. A stock solution of 80% sorbitol (w/v) was prepared and adjusted to generate 10% and 15% (w/v) concentrations in a final volume of 120 μL. For ethanol, 10% (v/v) and 15% (v/v) was used to induce ethanol stress. Temperature was adjusted to either 30°C, 35°C, or 40°C and data was taken at 15 min intervals for 96 hours for 30°C and 35°C, and 24 hours for assays at 40°C. Assays at 40°C were limited in terms of time due to the effect of evaporation if measured for 96 hours. Strains were prepared for inoculation onto PM assay plates as follows. Glycerol stocks stored at −80°C were streaked onto YPD plates to obtain single colonies and incubated at 30°C for approximately 48 hrs. Two to three colonies from each strain were then patched on a fresh YPD plate and incubated overnight at 30°C. Cells were then inoculated into sterile water in 20 × 100 mm test tubes and adjusted to a transmittance of 62% (~5 × 10^6^ cells.ml^−1^) using sterile distilled water using turbidometer. Cell suspensions for the inoculums were then prepared by mixing 125 μl of these cells and 2.5 ml of IFY buffer™ (Biolog, USA) and the final volume adjusted to 3 ml using RO sterile distilled water, 90 μl of this mix was inoculated to each well in a Biolog 96-well plate. Anaerobic conditions were generated by placing each plate into a PM gas bag (Biolog, Hayward, CA, USA) and vacuum packed using an Audion VMS43 vacuum chamber (Audion Elektro BV, Netherlands).

The OmniLog reader photographs the plates at 15 min intervals to measure dye conversion, the pixel intensity in each well is then converted to a value reflecting metabolic output. After completion of the run, the data was compiled and exported from the Biolog software and compiled using Microsoft® Excel. In all cases, a minimum of three replicate PM assay runs were conducted and the mean values are presented.

Percentage redox signal intensity was calculated by dividing the redox signal intensity value under stress conditions divided by the redox signal intensity under non-stress conditions after 50 hrs incubation, except for thermal stress at 40°C, where this was calculated using the redox signal intensity values at 24 hours for control and stressed conditions.

Data were transformed according to [39]. In order to eradicate noise a smoothing parameter was employed by sequentially increasing the number of moving average data points until all negative slopes between points had disappeared. A sliding window average of 25 data points was used to smooth the transformed metabolic output curves in order to facilitate comparisons and analysis.

### R statistical computing environment

Data from the 48 hr time points were analysed using R version 3.01, platform x86_64-w64-mingw32/x64 [59], data converted into comma delimited files and run on a R workspace, RGui 64 bit is a free to use software for statistical analysis package http://cran.r-project.org/bin/windows/base/. This package was used to compare sugar utilisation of *Saccharomyces* spp. yeast strains.

### Spot plate assays

Spot plate tests were performed according to [60] with modifications. Cells were grown overnight in yeast peptone broth (YPD) at 30°C with orbital shaking at 150 rpm [61]. One mL of culture (OD_600_ = 1.0) was centrifuged for 2 minutes at 17, 000 x g in a swinging rotor centrifuge operated at 4°C. The resulting pellet was washed three times using sterile distilled water and re-suspended in 100 μL of sterile distilled water. Next, the re-suspended cells were diluted to an initial OD_600_ of 1, serially diluted, and a 5 μL aliquot from each 10-fold dilution was spotted onto agar plates containing 0.67% YNB + 6% glucose and incubated at 35°C and 40°C for 3 days, no inhibitory compounds were added.

### Confirmation of phenotypic microarray results using mini fermentation vessels

Fermentations were conducted in 180 mL mini-fermentation vessels (FV). Cryopreserved yeast colonies were streaked onto YPD plates and incubated at 30°C for 48 hrs. Colonies of yeast strains YPS606, and DBVPG1583 were used to inoculate 20 ml of YPD broth and incubated in an orbital shaker at 30°C for 24 hrs. These were then transferred to 200 mL of YPD and grown for 48 hrs in a 500 ml conical flask shaking at 30°C. Cells were harvested and washed three times with sterile RO water and then re-suspended in 5 ml of sterile water. For control conditions, 1.5 × 10^7^ cells.mL^−1^ were inoculated in 99.6 ml of medium containing 4% glucose, 2% peptone, 1% yeast extract with 0.4 ml RO water. For stress conditions, 1.5 × 10^7^ cells.mL^−1^ were incubated in 99.6 ml of medium containing 4% glucose, 2% peptone, 1% yeast extract with 10 mM acetic acid, 5 mM formic acid, 5 mM levulinic acid, 5 mM furfural, 5 mM HMF and 5 mM vanillin. Volumes of media were adjusted to account for the addition of the inhibitory compounds (~400 μL) to ensure that all fermentations began with the same carbon load.

Anaerobic conditions were prepared using a sealed butyl plug (Fisher, Loughborough, UK) and aluminium caps (Fisher Scientific). A hypodermic needle attached with a Bunsen valve was purged through rubber septum to facilitate the release of CO_2._ All experiments were performed in triplicate and weight loss was measured at each time point. Mini-fermentations were conducted at 35°C, with orbital shaking at 200 rpm.

### Hierarchical clustering analysis of fermentation stress conditions

The hierarchical clustering algorithm used is based closely on the average-linkage method of Sokal and Michener, 1958, the object of this algorithm is to compute a dendrogram that assembles all elements into a single tree [2,62]. The matrix is scanned to identify the highest value (representing the most similar pair of strains) in comparison with the reference yeast strain *S. cerevisiae* S288C. Gene Cluster 3.0 was used to construct matrices [63] which are compatible with TreeView for production of representative dendrograms [63,64].

## Abbreviations

LCM: Lignocellulosic material; PM: Phenotypic microarray; NS: Nutrient supplement.

## Competing interests

The authors declare that they have no competing interest.

## Authors’ contributions

TW and DG equally contributed to work including generating data and writing the manuscript. MM and GL helped in designing experiments. GL donated some of the hybrid strains and EL donated the *sensu stricto* yeast. YC and AH helped in collecting data and MM helped in processing it. MM, GT, TP, EL and KS have proof read the manuscript. All authors read and approved the final manuscript.

## Supplementary Material

Additional file 1: Figure S1 Effect of temperature on growth of *S. uvarum*. Cells were grown on YNB with 6% (w/v), under aerobic conditions at a variety of incubation temperatures for 36 hours. *S. cerevisiae* (S288C) was added as a reference strain. **Figure S2.** Comparison of sensitive (DBVPG1853) and tolerant (YPS606) *S. cerevisiae* strains to 15% sorbitol, 10% ethanol, 35°C and 40°C, 25 mM acetic acid, 10 mM formic acid, 10 mM levulinic acid, 10 mM HMF, 10 mM furfural and 10 mM vanillin individually using phenotypic microarray analysis. Data expressed as% RSI of the unstressed wells. **Figure S3.** Number of *Saccharomyces* strains identified as more tolerant than the parent strains utilised to produce hybrids under sorbitol, formic acid and temperature stress, metabolic output for *S. cerevisiae* (Y12), *S. cerevisiae* (YPS128), *S. cerevisiae* (Y55) and *S. kudriavzevii* (IFO1803) for 10% (w/v) sorbitol, 5 mM formic acid and 35°C. Click here for file

Additional file 2 **Phenotypic microarray analysis (redox signal intensity) for *Saccharomyces *spp.** at 25 hour time point, data in the file is tabulated in the following order 1- Strain details and background information, 2 - Percentage redox signal of the control values for all stress conditions, 3 - Ranking of the strains for osmotic stress, 4 - Ranking of the strains for ethanol stress, 5 - Ranking of the strains for thermal stress- 35'C, 6 - Ranking of the strains for thermal stress- 40'C, 7 - Ranking of the strains for fermentation stress, 8 - Ranking of the strains for acetic acid, 9 - Ranking of the strains for formic acid, 10 - Ranking of the strains for levulinic acid, 11 - Ranking of the strains for HMF, 12 - Ranking of the strains for furfural, 13 - Ranking of the strains for vanillin, 14 - Ranking of the strains for vanillin and HMF, 15 - Ranking of the strains for the inhibitors, 16 - Ranking of the strains for all fermentation stress. The data shown is an average of triplicate values. Click here for file
