# Supplementary material for: Phenotypic characterisation of Saccharomyces spp. yeast for tolerance to stresses encountered during fermentation of lignocellulosic residues to produce bioethanol
Source: Microb Cell Fact. 2014 Mar 27;13:47. doi: 10.1186/1475-2859-13-47 (PMC3986927; doi:10.1186/1475-2859-13-47)
Supplement: Additional file 1: Figure S1 — Effect of temperature on growth of S. uvarum. Cells were grown on YNB with 6% (w/v), under aerobic conditions at a variety of incubation temperatures for 36 hours. S. cerevisiae (S288C) was added as a reference strain. Figure S2. Comparison of sensitive (DBVPG1853) and tolerant (YPS606) S. cerevisiae strains to 15% sorbitol, 10% ethanol, 35°C and 40°C, 25 mM acetic acid, 10 mM formic acid, 10 mM levulinic acid, 10 mM HMF, 10 mM furfural and 10 mM vanillin individually using phenotypic microarray analysis. Data expressed as% RSI of the unstressed wells. Figure S3. Number of Saccharomyces strains identified as more tolerant than the parent strains utilised to produce hybrids under sorbitol, formic acid and temperature stress, metabolic output for S. cerevisiae (Y12), S. cerevisiae (YPS128), S. cerevisiae (Y55) and S. kudriavzevii (IFO1803) for 10% (w/v) sorbitol, 5 mM formic acid and 35°C. [file 1475-2859-13-47-S1.pptx]

## Slide 1
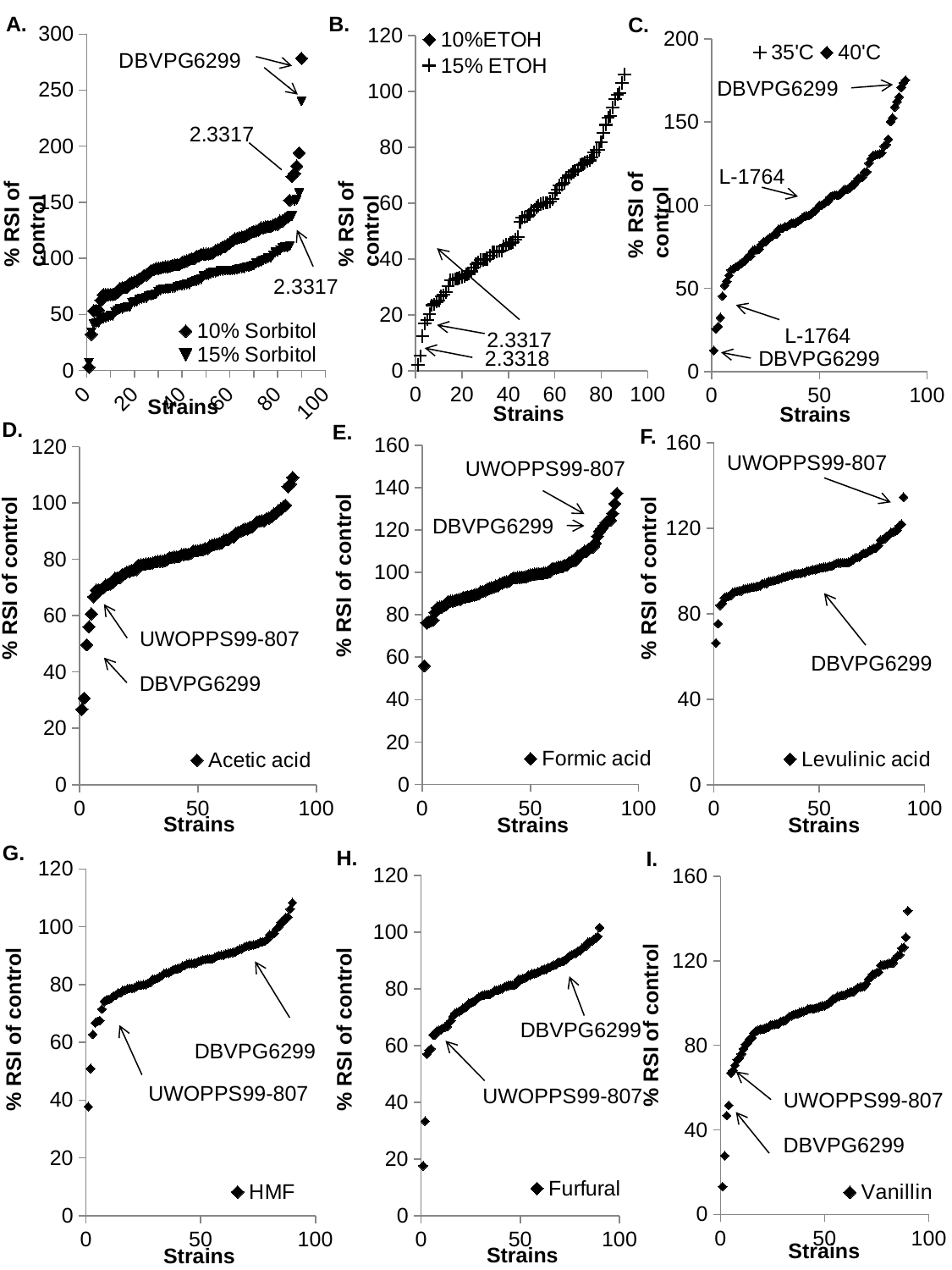

### Chart
| Category | 10% Sorbitol | 15% Sorbitol |
|---|---|---|A.
B.
C.
### Chart
| Category | 10%ETOH | 15% ETOH |
|---|---|---|
### Chart
| Category | 35'C | 40'C |
|---|---|---|DBVPG6299
% RSI of control
% RSI of control
% RSI of control
L-1764
2.3317
L-1764
2.3317
2.3318
DBVPG6299
D.
% RSI of control
### Chart
| Category | Levulinic acid |
|---|---|E.
F.
### Chart
| Category | Formic acid |
|---|---|
### Chart
| Category | Acetic acid |
|---|---|% RSI of control
UWOPPS99-807
UWOPPS99-807
% RSI of control
DBVPG6299
UWOPPS99-807
DBVPG6299
DBVPG6299
Strains
Strains
Strains
G.
H.
I.
### Chart
| Category | HMF |
|---|---|
### Chart
| Category | Furfural |
|---|---|
### Chart
| Category | Vanillin |
|---|---|% RSI of control
% RSI of control
% RSI of control
DBVPG6299
DBVPG6299
UWOPPS99-807
UWOPPS99-807
UWOPPS99-807
DBVPG6299
Strains
Strains
Strains

## Slide 2
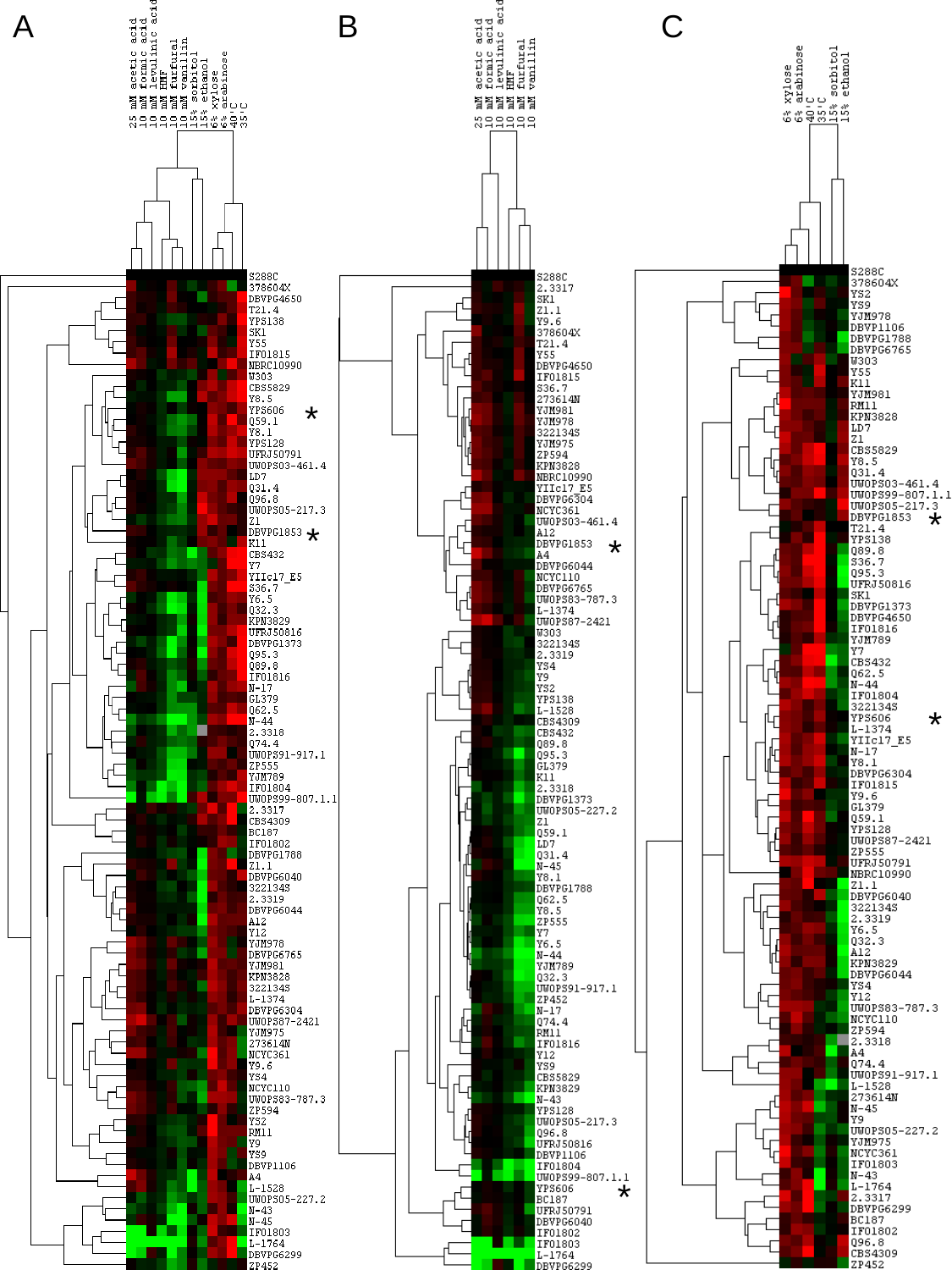

A
B
C
*
*
*
*
*
*

## Slide 3
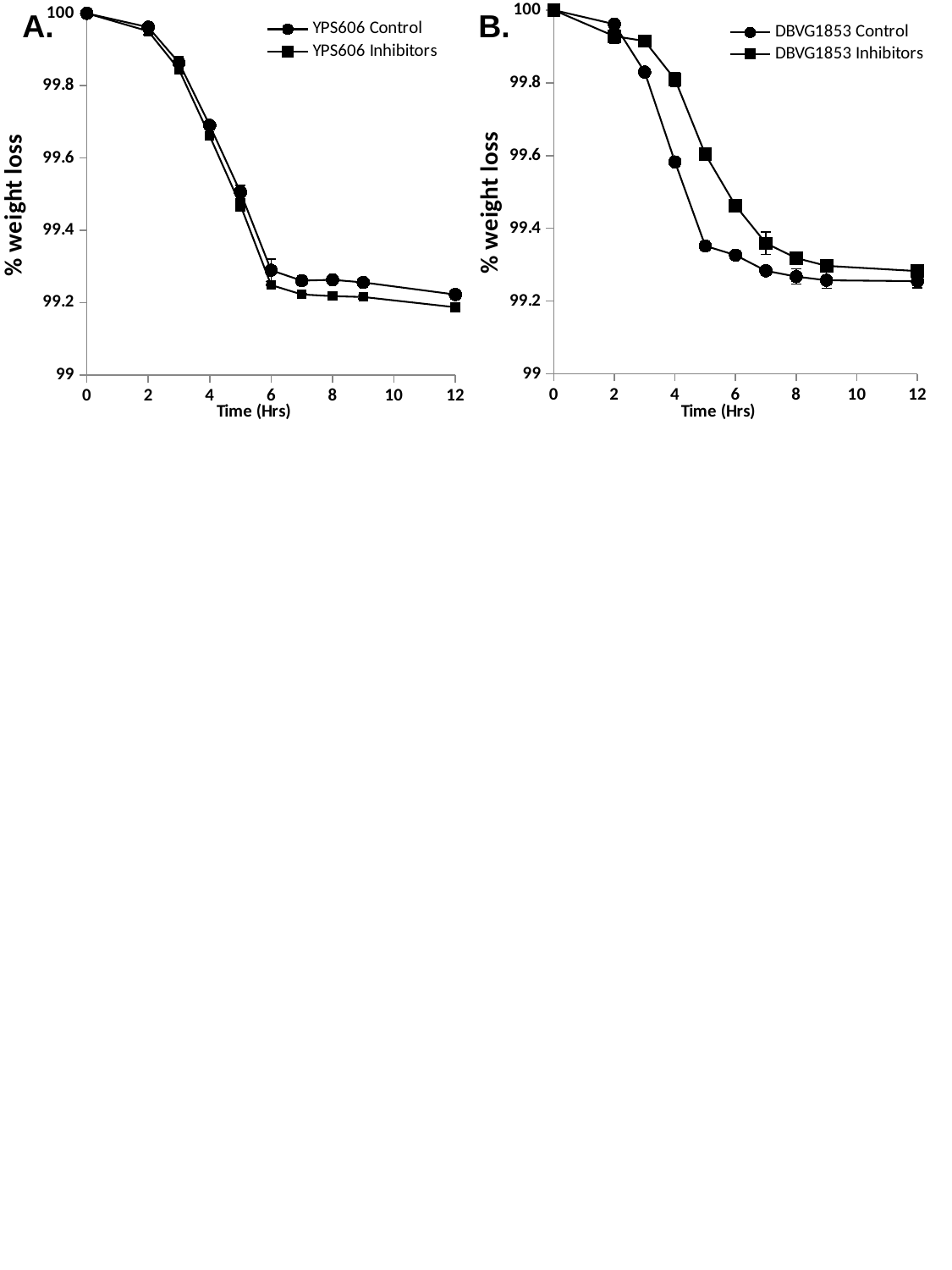

### Chart
| Category | YPS606 Control | YPS606 Inhibitors |
|---|---|---|A.
B.
### Chart
| Category | DBVG1853 Control | DBVG1853 Inhibitors |
|---|---|---|

## Slide 4
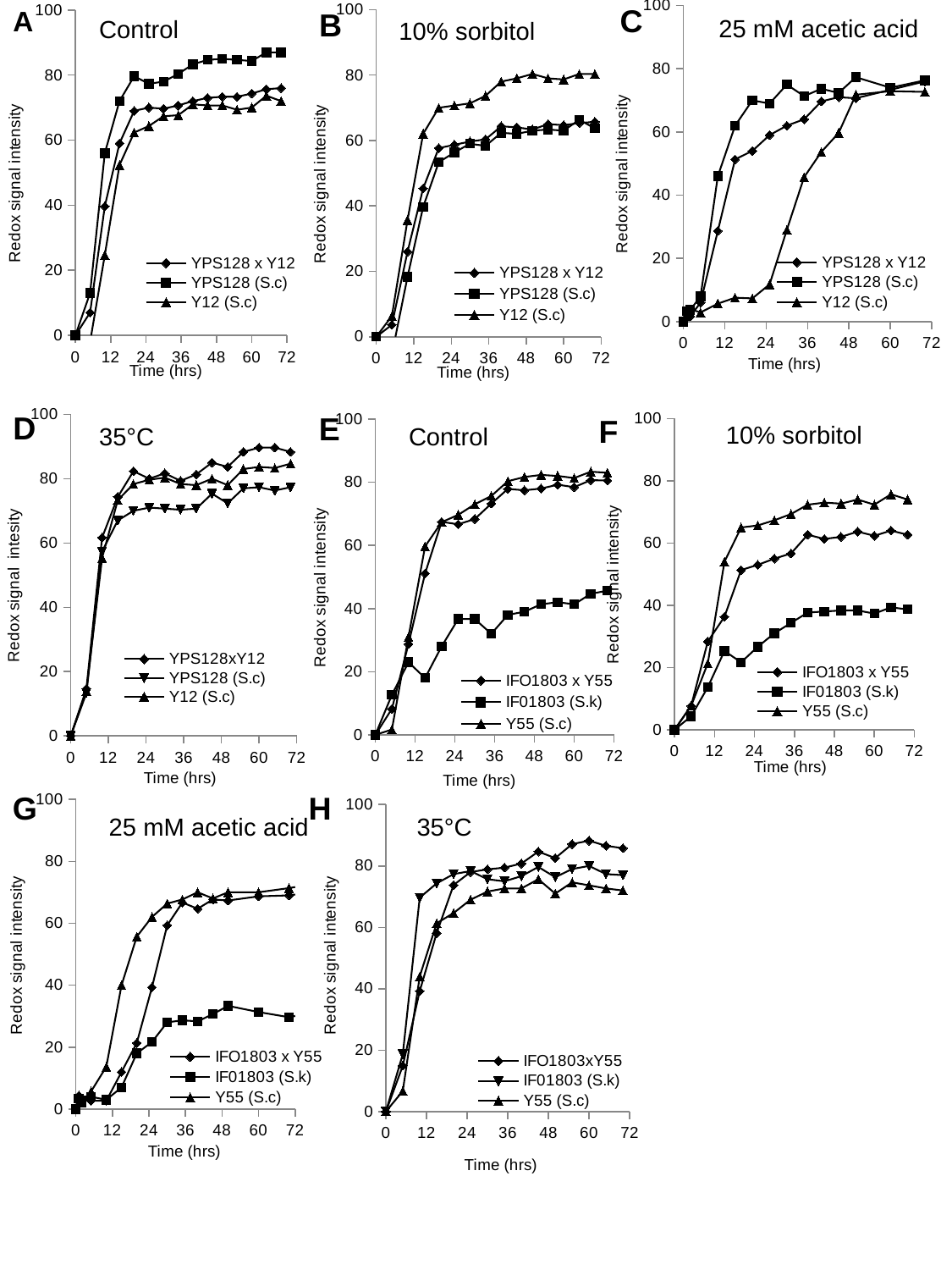

### Chart
| Category | YPS128 x Y12 | YPS128 (S.c) | Y12 (S.c) |
|---|---|---|---|
### Chart
| Category | YPS128 x Y12 | YPS128 (S.c) | Y12 (S.c) |
|---|---|---|---|
### Chart
| Category | YPS128 x Y12 | YPS128 (S.c) | Y12 (S.c) |
|---|---|---|---|25 mM acetic acid
Control
10% sorbitol
### Chart
| Category | YPS128xY12 | YPS128 (S.c) | Y12 (S.c) |
|---|---|---|---|
### Chart
| Category | IFO1803 x Y55 | IF01803 (S.k) | Y55 (S.c) |
|---|---|---|---|
### Chart
| Category | IFO1803 x Y55 | IF01803 (S.k) | Y55 (S.c) |
|---|---|---|---|10% sorbitol
35°C
Control
### Chart
| Category | IFO1803 x Y55 | IF01803 (S.k) | Y55 (S.c) |
|---|---|---|---|
### Chart
| Category | IFO1803xY55 | IF01803 (S.k) | Y55 (S.c) |
|---|---|---|---|35°C
25 mM acetic acid
